# Anti-Nutritional Factors of Plant Protein Feeds for Ruminants and Methods for Their Elimination

**DOI:** 10.3390/ani15081107

**Published:** 2025-04-11

**Authors:** Zhiyong Yan, Zixin Liu, Chuanshe Zhou, Zhiliang Tan

**Affiliations:** 1 Hunan Provincial Key Laboratory of Animal Nutritional Physiology and Metabolic Process, National Engineering Laboratory for Pollution Control and Waste Utilization in Livestock and Poultry Production, Institute of Subtropical Agriculture, Chinese Academy of Sciences, Changsha 410125, China; yanzhiyong23@mails.ucas.ac.cn (Z.Y.); lzx@isa.ac.cn (Z.L.); zltan@isa.ac.cn (Z.T.); 2 College of Advanced Agricultural Sciences, University of Chinese Academy of Sciences, Beijing 100049, China; 3 State Key Laboratory of Forage Breeding-by-Design and Utilization, National Engineering Laboratory for Pollution Control and Waste Utilization in Livestock and Poultry Production, and Hunan Provincial Key Laboratory of Animal Nutritional Physiology and Metabolic Process, Institute of Subtropical Agriculture, Chinese Academy of Sciences, Changsha 410125, China; 4 Yuelushan Laboratory, Changsha 410125, China

**Keywords:** plant protein feeds, anti-nutritional factors, ruminants, microbial degradation

## Abstract

In recent years, the rapid development of the ruminant feeding industry and the limited availability and rising prices of traditional protein feed ingredients have renewed the focus on protein feeds in ruminant diets. Plant protein feeds are a core component of protein feeds for ruminants; however, the utilisation of both conventional and non-conventional plant protein feeds is limited by the presence of anti-nutritional factors (ANFs). In order to maximise the use of plant protein feeds and to promote their application in ruminant production, it is important to have a comprehensive understanding of the types and nature of their ANFs, their anti-nutritional mechanisms, and current effective methods of eliminating ANFs. Therefore, the types, anti-nutritional mechanisms, and elimination methods of ANFs in major plant protein feeds for ruminants are initially summarised in this review, which provides a reference for anti-nutritional factor elimination and the production of full-price compound feeds for ruminants.

## Simple Summary

Ruminants are closely related to human life because they are able to convert plants into nutrients available to humans, including meat and milk. In modern agriculture, ruminant feeds include protein feeds, energy feeds, roughages, and additive feeds, where protein feeds can be categorised into plant proteins, animal proteins, microbial proteins, and non-protein nitrogen, according to the feed source. Only more plant protein feeds such as soybean meal can maintain a higher level of ruminant feeding to meet people’s demand for meat and milk; however, some anti-nutritional factors such as gossypol and tannins present in the plants themselves can affect the utilisation efficiency of ruminant feeds and even the health of ruminants, and it has become an imperative task to improve and solve this issue. Different plants contain different kinds of anti-nutritional factors, and different anti-nutritional factors have different physicochemical properties, so the corresponding treatment methods are also different. In order to minimise the impact of anti-nutritional factors in plants on ruminant production, we have to find out the distribution of anti-nutritional factors in different plants, the physicochemical properties of different anti-nutritional factors, and the most reasonable methods to eliminate them.

## 1. Introduction

In recent years, the price of feed raw materials has increased, the feed cost of farming has increased by 70~80%, and the increase in meat consumption requires more protein feed to maintain the level of animal production [1]. The overall shortage of feed raw materials and the expensive price of high-quality protein feed resources have become the main problems limiting the efficient farming of ruminants, and improving the digestive and absorptive efficiency of the feeds in ruminants is an effective way to alleviate the above problems. The feed formulation for ruminants is a complex and delicate process, aiming to fully satisfy their nutritional needs for growth, development, health maintenance, and performance enhancement. This integrated system consists of the following four main feed categories: protein feeds, energy feeds, roughages, and feed additives, each of which carries specific nutritional and physiological functions that together support a healthy feeding system for ruminants. Protein feeds are the core component of ruminant feeds. Protein feeds can be divided into plant protein, animal protein, microbial protein, and non-protein nitrogen according to the feed source. Plant protein feed sources mainly include all kinds of oilseed crops after the oil extraction of the remaining cake meal, including soybean meal (SBM), rapeseed meal (RSM), cottonseed meal (CSM), peanut meal, sunflower kernel meal, flax kernel meal, and so on. RSM, CSM, and other types of miscellaneous meal follows the ‘SBM reduction in feed’ trend. The application of the proportion of plant protein feed sources increased year by year, but it contains a variety of anti-nutritional components ANFs to limit the various types of miscellaneous meal as well as the application of SBM [2]. When livestock and poultry consume excessive amounts of ANFs, it not only affects the digestibility and absorption of nutrients, but it may even have adverse effects on growth and health [3,4]. For example, free gossypol, glucosinolates, trypsin inhibiting factor, antigenic proteins, and phytohemagglutinins present in cottonseed will inhibit the absorption of amino acids, and RSM contains a large amount of glucosinolates, tannins, phytic acid, erucic acid, and other ANFs [5,6]. At the same time, the nutrient composition of various types of miscellaneous meals is also defective, for instance, the lysine and threonine content of RSM is lower than that of SBM, and the digestibility in ruminants is significantly lower than that of SBM [7]. In order to improve the utilisation efficiency of plant protein feeds and their processing by-products, the inactivation of ANFs in them is a critical step. Currently, a number of different methods have been investigated to eliminate or inactivate these ANFs, mainly covering the following three techniques: physicochemical treatment, enzymatic treatment, and microbial decomposition [8]. This paper comprehensively reviews the classification, anti-nutritional mechanisms, and mitigation strategies of ANFs in plant-based protein feeds for ruminants. By synthesising current knowledge, this work aims to provide actionable insights for optimising feed formulations, enhancing nutrient utilisation, and advancing the development of balanced, high-performance compound feeds. Ultimately, this review serves as a foundational resource to guide future research and industry practices in sustainable ruminant nutrition.

## 2. Overview of Plant Protein Feeds and ANFs

Plant protein feed sources mainly include the cake meal remaining after the oil extraction from various oilseed crops, including SBM, RSM, CSM, and so on. They have great potential as a protein source for ruminant protein feeds due to their richness in crude protein, amino acids, and good availability [9]. These plant protein feeds contain a variety of ANFs, such as protease inhibitors, antigenic proteins, and gossypol, which limit the efficiency of protein and carbohydrate utilisation in plant protein feeds and adversely affect the growth and reproduction of animals. The content and physicochemical properties of different ANFs in different feeds are varied, so the corresponding elimination methods are also different [10].

### 2.1. ANFs in SBM

SBM is the most commonly used plant-based protein feed in various animal feeds because of its high yield, high crude protein content, good quality, reasonable composition of essential amino acids, and high digestibility [3]. However, soybean also contains ANFs such as protease inhibitors, soybean antigens, soybean agglutinin, etc., which reduce its nutritional value.

#### 2.1.1. Soybean Antigenic Protein

The main ANFs in soybeans are antigenic proteins, which are immunoreactive and thermostable, may cause allergic reactions in animals, and may affect their productive performance [11]. Soybean antigenic proteins can be classified into four categories according to the centrifugal sedimentation coefficient as follows: 2 S, 7 S, 11 S, and 15 S, with the highest proportion of soybean antigenic proteins being 11 S at approximately 40% [12]. The main components of soybeans that trigger the immune response of animals are *β*-accompanied by soybean globulin (7 S) and soybean globulin (11 S), with the 7 S protein having the greater immunoreactivity, and 11 S proteins consisting of six subunits [13]. Each of these is connected to a disulfide bond, subunits, connecting basic and acidic peptide chains via disulfide bonds [11].

#### 2.1.2. Protease Inhibitors

Protease inhibitors are capable of hindering the function of a variety of proteases, such as trypsin, pepsin, and coagulation factors, etc. There are numerous types of protease inhibitors in nature, among which, the main ones contained in SBMs are trypsin inhibitors (TIs), classified into Bowman-Birk inhibitors (BBIs) and Kunitz inhibitors (KTIs) [14]. The KTI has a variable structure containing four cysteine residues, which form two disulfide bonds, a structure that makes the KTI easy to destroy when heated. The BBI contains a higher number of cysteines and seven disulfide bonds, and it has multiple reactive groups that can inhibit the activity of multiple proteases simultaneously. Although the content of TIs in soybean is only 2% of the total mass (about 0.6% for BBI and 1.4% for KTI), they account for 40% of the total anti-nutritional effect of all ANFs in soybean [4].

#### 2.1.3. Soybean Agglutinin

Soybean agglutinin (SBA), as the name indicates, is a class of ANFs that promotes erythrocyte aggregation and is a major anti-nutrient in soybean, which is heat-insensitive, stable, and undamaged. In SBM, the concentration of SBA is typically 3% [15]. SBA impairs animal growth by interfering with the digestion and absorption of nutrients in the gastrointestinal tract. In addition, one of the most important characteristics of SBA considered to be ANFs is the remarkably high resistance and stability to protein hydrolysis over a large physiological pH range [16]. When SBA is ingested by livestock, SBA specifically binds to glycoproteins on the surface of small intestinal epithelial cells, thereby adversely affecting the animal’s intestinal epithelial cells and immune function. Some studies based on intestinal porcine epithelial cell lines (IPEC-J2) have shown that SBA exerts its toxic effects on the intestine through a variety of apoptosis-related pathways [17]. Other studies have shown that SBA have an effect on growth performance in young ruminants, but not in adult ruminants [18]. SBA may be degraded by rumen microorganisms.

#### 2.1.4. Tannins

Tannins are water-soluble natural polyphenolic compounds, mainly found in the shell of soybeans, which can be divided into hydrolysed tannins (HTs) and condensed tannins (CTs). CTs cannot be hydrolysed and can react with amylase, trypsin, and their substrates, thus decreasing the utilisation rate of carbohydrates and proteins, and affecting the intake of the animal [19]. Tannins not only reduce the digestibility of feed proteins, but also affect the intestinal micro-ecosystem. When tannins are increased in feeds, they significantly reduce total gas production and the concentration of ammonia and volatile fatty acids in the intestinal tract, and they increase the number of viable bacteria in the enterococci and coliform flora [20].

#### 2.1.5. Phytic Acid

Phytic acid (PA), chemically known as inositol hexakisphosphate, exists mainly in the form of phosphorus storage from seeds of cereal and oilseed crops, and its molecular structure contains an inositol ring and six phosphoric acid groups. PA is present not only in legume feeds, but also in rapeseed and its cake meal [21]. The PA molecule consists of six phosphate groups, which can interact with metal ions and proteins to produce insoluble complexes, which are not easily digested and absorbed by animals, reducing the nutritional value of the feed and affecting its palatability, which has a negative impact on the healthy growth of animals [22].

#### 2.1.6. Non-Starch Polysaccharides

SBM contains many non-starch polysaccharides (NSPs), of which *β*-glucan, arabinoxylan, and cellulose are the main components, usually accounting for less than 10% of its weight [23]. The high proportion of ether bonds in lignin makes it more difficult to degrade than cellulose and hemicellulose, and it requires 150–400 °C to break [24]. Degradation of NSPs in ruminants can occur in the rumen, small intestine, and large intestine, but the rumen is the most important degradation site for feed NSPs, most of which are rapidly degraded mainly by rumen microorganisms. It has been shown that the ingestion of NSPs can have a systemic viscosity effect, which can inhibit glucose absorption or form a physical barrier to inhibit starch digestion by amylase and reduce its digestion rate [25,26,27].

#### 2.1.7. ANFs in Miscellaneous Meals

Miscellaneous meals contain high levels of protein and a variety of vitamins and minerals, but their amino acid ratios are unbalanced, and their use in ruminant feeds is limited by the presence of free lintol, thioglucosides, PA, and other ANFs in miscellaneous meals [24].

#### 2.1.8. Gossypol

Gossypol is a phenolic substance that is particularly abundant in the seeds of the cotton plant, with the molecular formula C_50_H_50_O_8_. In CSM, gossypol exists in both free and bound forms, and the total amount of gossypol in both forms is referred to as total gossypol [28,29]. The World Health Organisation (WHO) in 2018 established a maximum threshold of 450 mg/kg for the content of free gossypol (FG) in CSM protein products [30]. Gossypol damages the gastrointestinal mucosa of animals and the reproductive capacity of males, and tolerance to gossypol varies in different animals, with pigs having a low tolerance and broilers and laying hens having a relatively high tolerance [29]. Ruminants have a better tolerance to gossypol due to their special digestive system, and it was found that CSM can replace SBM as the main protein source for beef cattle and goats, without affecting their growth and slaughter performance [31]. However, gossypol has toxic effects on the reproductive system of ruminants. Tiago et al. collected testicular samples from young male goats after feeding them with gossypol for 95 days, analysed them, and found that there was a significant decrease in the concentration of testosterone after feeding gossypol, as well as higher total sperm defects, poor sperm motility, and a high rate of lesions [32]. This indicates that the intake of gossypol in ruminants used for breeding is unsafe.

#### 2.1.9. Glucosinolates

There are many glucosinolates in RSM that are decomposed by mustard enzymes to produce oxazolidinethione (OZT), isothiocyanate (ITC), and nitrile, with OZT being the main toxin in RSM, and it is more toxic to the thyroid gland. OZT is the main toxin in RSM and has a strong toxic effect on the thyroid gland [33]. ITC can damage the mucous membranes of the gastrointestinal tract of animals or cause substantial damage to the lungs and kidneys due to its own irritant properties [34]. Nitrile is the most toxic of the glucosinolates decomposition products, and prolonged or large intake can damage the liver and kidneys of animals [35]. Tolerance to glucosinolates varies among animals, being higher in ruminants and lower in pigs and poultry [36,37]. The treatment of CSM and RSM by physical, chemical, and biological methods can reduce the content of ANFs and improve the digestion and absorption of nutrients in animals [38]. The anti-nutritional mechanisms of ANFs in plant protein feeds are shown in Table 1.

## 3. Advances in ANF Elimination Methods

Currently, there are many techniques to eliminate ANFs in plants, such as high temperature and pressure, fermentation, etc., but they can be broadly categorised as physical, chemical, and biological methods [56]. Physical methods completely deactivate ANFs through high temperature, high pressure, radiation, etc. The chemical method involves adding chemical reagents (e.g., CuSO_4_ and H_2_O_2_, etc.) to feeds containing ANFs under appropriate conditions to inactivate ANFs through chemical reactions. Biological methods, on the other hand, include enzymatic degradation by enzymes and microbial fermentation to inactivate ANFs or degrade them into other small molecules.

### 3.1. Physical Methods

For heat-stable ANFs, such as PA and tannins, physical methods such as peeling and crushing can be used to reduce their content. For example, soybean can remove most of the tannins and reduce PA content by removing the outer hulls [58]. For thermally unstable ANFs, such as agglutinins and protease inhibitors, a common method is heating [59].

Heating methods include dry heat treatments (e.g., baking, microwave heating, and infrared irradiation) and moist heat treatments (e.g., boiling, autoclaving, and extrusion heating), which are only suitable for thermally unstable ANFs. They are not effective for thermally stable ANFs, such as PA, cyanogenic compounds, and oligosaccharides. RSM heated at 100 °C for 5 min or 2 h increased the removal of glucosinolates from 24% to 95% but protein digestibility decreased from 79% to 71% [60]. Duodu et al. used soaking and autoclaving techniques, leading to a reduction in FG levels by 34.48% and 27.59%, respectively, as well as a significant reduction in PA [61]. Water immersion also removed soluble NSPs [62]. In addition to this, studies have also reported the elimination of NSPs using electron beam (EB) irradiation methods [63]. The method of electron radiation also effectively eliminates FG and total gossypol in CSM. Bahraini et al. compared the effects of EB and gamma ray (GR) irradiation treatments at doses of 10, 20, and 30 kGy on the chemical composition, protein quality, and protein digestibility of CSM, significantly reducing FG and total gossypol content in CSM in a dose-dependent manner compared with unirradiated CSM. In addition, ER irradiation induced a greater decrease in FG and total gossypol content than GR irradiation [64].

The processing of feeds also affects the degradation or deposition of ANFs over time. The first step in feed production is comminution, which also destroys the fibrous structure of the raw material and increases the solubility of NSPs, making them available to digestive enzymes. Extrusion is a high-temperature, short-duration process that combines several processes, including heat and mass transfer, mixing, shearing, particle size reduction, melting, texturizing, caramelising, and moulding. Extrusion is also a very effective method to inactivate amylase inhibitors, trypsin, pancreatic rennet, and SBA activity without altering the protein levels in the food [65]. In general, the moisture content and composition of raw materials, barrel temperature, and extruder feed rate are the most important factors affecting the extrusion process in terms of reducing the amount of ANFs [66]. In another study, the effectiveness of extrusion processing was compared with conventional non-thermal processing methods such as dehulling, soaking (in double deionised water at 30 °C for 12 h), and germination (at 25 °C for 72 h) in an attempt to reduce ANFs in broad beans and kidney beans. The results showed that germination for 72 h resulted in a significant reduction in phytates in broad beans (60.8%) and kidney beans (30.2%), whereas extrusion alone resulted in a reduction in phytates by about 26.7% and 21.4% in broad beans and kidney beans, respectively [67].

### 3.2. Chemical Methods

Chemical methods refer to the addition of acids, bases, alcohols, ammonia, and special substances to react with ANFs or purify ANFs by extraction. Chemical methods can deactivate ANFs by decomposing them, neutralising them, dissolving them, binding them, destroying the structure of the ANFs, or breaking the bonds that maintain the structure. For example, the treatment of SBM with ascorbic acid, CuSO_4_, FeSO_4_, and urea can inactivate TIs, and the addition of FeSO_4_ and CuSO_4_ to RSM results in a glucosinolates detoxification rate of up to 85.2%, which is less costly, but also suffers from poor palatability, severe nutrient loss, and some contamination [68].

Barraza et al. found that the addition of 0.5%, 1%, and 2% calcium hydroxide to CSM led to a reduction in FG by 21.25%, 28.15%, and 40.52%, respectively, but the use of calcium hydroxide often reduces the bioactivity and detoxification efficiency of the vitamins in the feed [69]. In addition, supercritical CO_2_ extraction and solvent extraction were more effective in removing FG. Bhattacharjee et al. used supercritical fluid extraction with CO_2_ to extract oil from CSM and found that when the extraction parameters were set at a pressure higher than 550 bar, a temperature range of 70–80 °C, and an extraction time of 2–3 h, it was possible to maximise the oil yield while minimising FG extraction [70]. Pelitire et al. used acetone and ethanol as solvents to remove FG from CSM and found that both solvents were effective in reducing the total FG level in CSM to between 5% and 10% of its initial value, with the rate of FG removal being much faster via ethanol extraction than via acetone extraction [71]. It is suggested that the process of supercritical CO_2_ extraction and solvent extraction can be used to produce CSM with low FG.

The chemical method is convenient in operation, has a good inactivation effect, and saves equipment and energy, but the chemical reagents may remain in the raw materials and need to be cleaned and dried after treatment, which not only increases the production cost, but it may also cause pollution to the environment. This limits the use of chemical methods in feed processes.

### 3.3. Biological Methods

#### 3.3.1. Enzymatic Method

The enzymatic method utilises enzyme preparations such as protease, NSPs enzyme, phytase, and tannase to degrade ANFs and thus reduce their content. Since the implementation of China’s policy of banning antibiotics in feed in 2020, enzyme preparation has been more and more widely used in the feed industry, which not only serves to eliminate ANFs in feed ingredients but also solves the problem of insufficient endogenous enzymes in young animals, promotes the secretion of endogenous enzymes, and improves the utilisation rate of proteins. Phytase is a critical feed additive for monogastric animals, as their gastrointestinal tracts naturally lack this enzyme. To enhance phytate hydrolysis in diets, exogenous phytase must be supplemented—primarily derived from fungal and bacterial strains [72]. Up to dozens more enzymes are known to catalyse PA cleavage, including phytase secreted by grains, intestinal mucosa, and gastrointestinal microbial communities and a large number of commercial preparations of phytase with modified properties, with the role of each phytase type and its maximum activity depending on the pH of the enzymatic environment [73]. On this basis, phytase is classified as either acidic or alkaline. Acidic phytase shows broad substrate specificity for metal-free phytates, while alkaline phytase is specific for metal-bound PA. Phytase promotes balanced animal mineral nutrition and significantly reduces phosphorus excretion from animal faeces with the addition of appropriate amounts of phytase to feeds, thus mitigating the ecological problem of phosphorus pollution [74].

#### 3.3.2. Microbial Degradation

Microbial fermented feed is a type of feed produced by the metabolism of microorganisms, which has the characteristics and effects of reducing the content of ANFs in raw materials, producing beneficial metabolites, and improving the digestion and absorption of feed nutrients by animals [75,76]. Fermentation is a dynamic process that converts complex substrates into simple compounds, and its products are mainly influenced by the microbial species, fermentation substrate characteristics, and fermentation parameters [77]. Probiotic species commonly used in fermented feeds include yeasts, bacilli, lactobacilli, and moulds [78]. Zheng et al. fermented SBM using *Bacillus sphaericus* and found that *Bacillus sphaericus* was able to degrade ANFs as well as change the microstructure of fermented SBM proteins to improve the nutritional quality of fermented SBM [79]. The fermentation of whole feeds is a more convenient way compared to the fermentation of single protein feed ingredients, but this type of fermentation results in a higher loss of amino acids such as lysine [80]. The fermentation process is catalysed by enzymes, and temperature is an important factor to ensure enzyme activity. Appropriate fermentation temperatures can help shorten the reaction stabilisation time, and Dujardin et al. showed that increasing the temperature of liquid fermented feeds from 15 °C to 30 °C reduced the time taken for the pH of the feeds to drop to 4.0 by six-fold [81]. As understood from the perspective of enzyme kinetics, increasing temperature accelerates microbial growth and metabolism [82]. In conclusion, microbial fermentation is a complex and dynamic fermentation process, and the fermentation end products are regulated by the interactions of its influencing factors. In order to obtain the desired fermentation products, the precise optimisation of fermentation conditions is imperative. The process of bacterial growth can decompose some of the ANFs, which significantly improves the efficiency of feed utilisation [83]. Protozoa and bacteria in the rumen of ruminants will compete in the decomposition of starch granules, slowing down the process of rumen fermentation and thus reducing problems such as acidosis [84].

The degradation of ANFs by microbial fermentation seems to be a promising and environmentally friendly method compared to physicochemical methods. The key to the degradation of ANFs by microbial degradation is the selection of bacterial strains. Many strains of bacteria (including Aspergillus, yeasts, and Bacillus) have been used in the microbial fermentation of plant protein feeds, since the fermentation process can secrete a variety of enzymes, including proteases, cellulases, and growth factors [85]. Improvement varies between strains, with some strains only improving roughage nutrient content and positively affecting the reduction in ANFs. Some strains affect only positively the improvement of enzyme activity in the gut, while other microorganisms can improve both [86]. For example, Bacillus sp. is able to degrade ANFs [79]. The effect of mixed-strain fermentation is generally better than that of single-strain fermentation, so mixed-strain fermentation is mostly used in practical production applications [75]. The microbial fermentation of plant protein feeds for ruminant production has also made progress with the widespread use of fermented feed technology. Wang et al. found that fermented SBM improved the rumen microecology of lactating dairy cows by increasing the number of copies of filamentous *Bacillus succinicus*, *Leptospira ruminantium*, and Prevotella [87]. Kim et al. reported that fermented SBM also reduced diarrhoea in calves [88]. Microbial fermentation not only improves the nutritional value and utilisation of feeds, but also improves the intestinal microbiota of animals, which plays a growth-promoting role [89].

Microbial fermentation can also destroy ANFs in miscellaneous meal. Khalaf et al. found that microbial fermentation significantly reduced FG in CSM by studying the effect of microorganisms screened during the solid-state fermentation of CSM. However, there were differences between different strains of bacteria, among which the optimal conditions for the reduction in FG content by Pseudohyphae tropicalis yeast fermentation were incubation for 48 h, incubation temperature of 30 °C, 55% moisture content of solid substrate, and pH value around 5.2. Under these optimised conditions, the crude protein and amino acid content of fermentation substrate was significantly increased [90]. Olukomaiya et al. used *Aspergillus soya* and *Aspergillus figlii* in a complex to ferment RSM and reduced the PA content from 27.06 mg/g to 22.13 mg/g [91]. Bacillus subtilis was able to produce extracellular enzymes, thereby promoting the degradation of acid detergent fibres (ADF) [86]. Lactobacilli were able to reduce the activity of trypsin inhibiting factor and reduce the content of ANFs in feeds [92]. After microbial fermentation, the content of ANFs decreased significantly [93,94]. Studies have shown that fermentation can remove 85% of ANFs and toxic substances from CSM and RSM [95]. However, the mechanism of the microbial fermentation of feeds for the removal of ANFs and the degradation of harmful substances in raw materials is not fully understood, and it may be related to microbial metabolism. Mi et al. showed that the addition of 19.04% fermented RSM to the diet of black goats significantly increased the abundance of propionate, butyrate, and volatile fatty acids (VFA) in the rumen, and it was also able to increase the number of beneficial bacteria in black goat rumen microbiota [96].

It is worth noting that microbial fermentation can not only reduce ANFs in miscellaneous meals, but it can also reduce costs and increase efficiency [97]. In addition, the process of feed fermentation also produces aromatic substances that enhance the flavour of feed ingredients to promote animal feeding, so it is more advantageous to use microbial degradation to treat ANFs. However, it is necessary to assess the safety of certain metabolites produced during fermentation to prevent them from posing any toxicity risks. The application of microbial degradation on different ANFs is shown in Table 2.

## 4. Prospects

**Figure 1:**
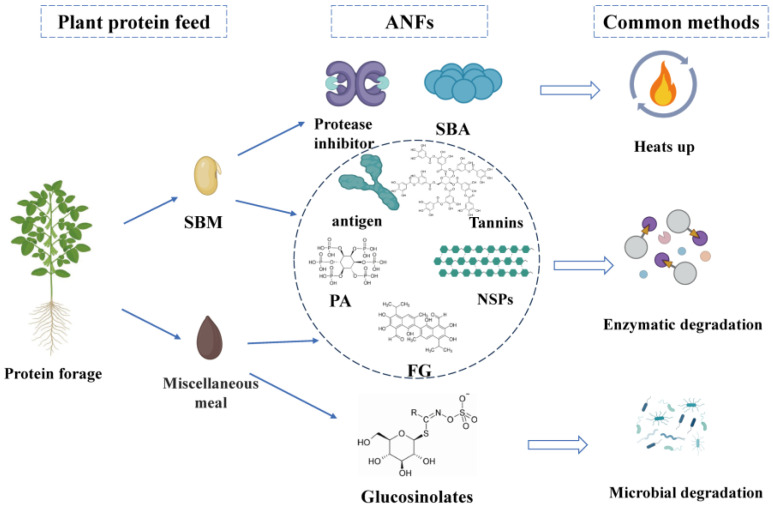
ANFs in plant protein feeds and common elimination methods (figure created in BioRender, https://biorender.com, accessed on 15 December 2024).

In the future, the chemical structure, biological activity, and mechanism of action of ANFs in feeds need to be deeply investigated to explore more efficient inactivation strategies. The currently available elimination methods have their obvious advantages and disadvantages. High-temperature treatments are particularly effective for unstable ANFs such as antigenic proteins and protease inhibitors, and dehulling also facilitates the removal of ANFs, such as tannins, which are mainly found in seed shells. While chemical methods have limited their use due to their toxicity and possible environmental stress, enzymes are more effective in removing stable ANFs and are now widely used in the feed industry. Microbial degradation is more effective than enzymatic degradation, but this depends on the type of microorganism, the amount added, and the fermentation conditions. In addition, certain emerging technologies such as gamma ray and irradiation have multiple benefits, such as the elimination of microbial and fungal contamination of SBM and miscellaneous meal while retaining protein and amino acid integrity, reduced energy consumption to reduce chemical reactions, etc. These should be further investigated in more detail in the future. At present, the specific mechanism of action of strains in the fermentation process has not been fully studied, and the effect of their application in production varies. Therefore, further research on the specific mechanism of action of different strains in the fermentation process, as well as the interaction effects between different strains, will help develop microbial fermented feeds that are more meaningful for ruminant nutrition. Nevertheless, the mechanism of microbial fermentation feed to reduce ANFs and toxic and harmful substances has not been clarified yet. Moreover, the preservation problem of strains used for fermentation has not been effectively solved, the ratio of fermentation of mixed bacteria needs to be studied in depth, and the speed of fermentation is difficult to be controlled. At the same time, the secondary fermentation of feeds will also bring about a certain amount of economic losses [106]. In the future, during the development and utilisation of microbial fermented feed, it is necessary to establish a special microbial fermented feed research database, improve the quality evaluation system, thoroughly study the action mechanism and advantages of microbial fermented feed, and promote the sustainable development of microbial fermented feed. Microbial fermented plant protein feeds have significantly improved the production performance of ruminants, but there is no significant improvement in the quality of ruminant products. Therefore, it will be of great significance to carry out the research work related to bacterial strain research, animal immunity, intestinal flora, nutrient metabolism, etc., in order to gain a deeper understanding of the mechanism of action of microbial fermented plant protein feeds, and to develop different strains of bacterial strains, different roughage, and different animal species fermented feeds according to the local conditions. Various types of miscellaneous meal due to the price advantage in the proportion of feed gradually increased. In order to solve the issue of the accumulation of gossypol, glucosinolates, tannins, and other ANFs, as well as other problems, biological fermentation and bacteria and enzyme synergistic treatment have been widely introduced into the production process, using various types of enzyme specificity and the high efficiency of ANFs for decomposition and transformation [2]. Biological methods have obvious advantages, as they do not need to use difficult to separate solvents, can significantly reduce costs, and can rely on the physiological activities of the bacterial colony to decompose various types of ANFs. Moreover, the bacterial colony can increase the protein content of the product after moderate reproduction to improve the composition of the product’s nutrient composition. However, the degradation rate of ANFs in the biological method process based on strain compounding is lower than that of the chemical method, and the synergistic treatment of bacteria and enzymes can improve the decomposition rate. However, there is no cost advantage compared with the chemical method, so the method urgently needs corresponding high enzyme-producing bacterial strains. The main ANFs of plant protein feeds and the common elimination methods are shown in Figure 1.

## 5. Conclusions

The types, anti-nutritional mechanisms and elimination methods of ANFs in major plant protein feeds for ruminants were initially summarised in this review, which provides a reference for antinutritional factor elimination and production of full-price compound feeds for ruminants. Currently, the variable quality of fermentation products from protein feed resources, the unclear association between fermentation effect and microbial strains, and the standardisation of the fermentation process remain the main challenges for the efficient utilisation of protein feed resources for ruminants. With the continuous research on fermentation technology, more effective methods of eliminating ANFs from plant protein feeds will be developed, which is important for alleviating the shortage of high-quality protein resources and enhancing the efficiency of the use of plant protein raw materials for ruminants.

## Figures and Tables

**Table 1:** Anti-nutritional mechanisms of ANFs in plant protein feeds.

ANFs	Anti-Nutritional Mechanisms	References
Soybean antigenic protein	It can cross the epithelium of the small intestine and enter the internal circulatory system, activating the immune response and promoting the production of specific antibodies by B-cells, leading to intestinal allergy and disruption of the intestinal barrier, which can lead to diarrhoea and a decline in production performance.	[12,39,40,41,42]
Protease inhibitors	It binds to the active site of trypsin to form a stable inhibitory complex, which renders trypsin and pancreatic rennet ineffective, thereby affecting the growth rate of the animal and possibly leading to abnormal pancreatic function.	[43,44]
SBA	SBA disrupts the intestinal flora environment and negatively affects the immune system by inhibiting the intestinal production of immunoglobulin A (IgA). SBA also acts by inducing apoptosis through the FAK signalling pathway.	[17,45,46]
Tannins	High levels of tannins react with salivary mucins to bring about astringency, which in turn reduces their intake and decreases the efficiency of nutrient transport by the intestinal mucosa. Tannins also affect the metabolism of indole compounds and androstenone in animals, which in turn affects the development of the secondary gonads.	[47,48,49]
Gossypol	FG inhibits cardiovascular diastole and contraction, hinders neurotransmitter transmission, complexes with amino acids, and reduces lysine utilisation efficiency. FG causes loss of electrical rhythm in animals, leading to heart failure. FG affects the function of various enzymes, alters the nature of cell membranes, forms a strong bond with metal ions, and interferes with the bioavailability of mineral elements.	[6,28,50,51]
NSPs	They have a complex structure that requires the simultaneous presence of multiple digestive enzymes for degradation, so they are less fermentable in the digestive tract. Insoluble cellulose consists of glucose linked by *β*-1,4-glycosidic bonds, which cannot interact with water after the formation of hydrogen bonds and is therefore degraded at a slower rate.	[25,26,52]
PA	PA has the ability to reduce the bioavailability of some essential minerals by chelating with divalent or multivalent metal ions (e.g., zinc, calcium, copper) to form insoluble PA salts. PA also directly affects the activity of digestive enzymes, such as proteases and amylases, which can inhibit protein breakdown and absorption during rumen fermentation in ruminants.	[53,54,55,56]
Glucosinolates	Glucosinolates hydrolysed to produce thiocyanate, isothiocyanate, and oxazolidinethione, which affects thyroid development and hormone secretion in animals.	[35,57]

**Table 2:** Application of microbial degradation to different ANFs.

ANFs	Microbiological Species	Experimental Methods	Effects	References
Soybean antigenic protein	*Bacillus licheniformis*	SBM fermented for 24 h	Soybean antigenic protein reduced by 25.5%	[98]
Soybean antigenic protein	*Bacillus cereus*	SBM fermented for 24 h	Soybean antigenic protein reduced by 68.14%	[99]
Protease inhibitors	*Planctomyces militaris*	De-oiled rice bran fermented for 3 d	TIs reduced by 24.8%	[100]
Protease inhibitors	*Bacillus Siamensis jl8*	SBM fermented for 24 h	TIs reduced by 95.01%	[79]
PA	*Bacillus subtilis*	Wheat flour fermentation for 24 h	PA content reduced by 55%	[101]
Glucosinolates	*Lactobacillus acidophilus*	RSM fermented for 25 d	Glucosinolates levels decreased by 67.81%	[102]
Tannins	*Saccharomyces cerevisiae*	SBM incubated at 28 °C for 48 h	Tannin degradation rate of 37.9%	[103]
NSPs	*Lactobacillus plantarum*	SBM fermented for 48 h	NSPs decreased by 39.15%	[104]
NSPs	*Bacillus licheniformis B4*	SBM fermented for 48 h	NSPs decreased by 30.20%	[98]
Gossypol	*Bacillus coagulans*	CSM fermented for 14 d	The detoxification efficiency of FG was 93.46%	[97]
Gossypol	*Lactobacillus agalactiae*	CSM anaerobically fermented for 5 d	The detoxification efficiency of FG was 80%	[105]

## Data Availability

Not applicable.

## Abbreviations

The following abbreviations are used in this manuscript:

ANFs	Anti-Nutritional Factors
SBM	Soybean Meal
RSM	Rapeseed Meal
CSM	Cottonseed Meal
TIs	Trypsin Inhibitors
BBI	Bowman-Birk Inhibitor
KTI	Kunitz Inhibitor
SBA	Soybean Agglutinin
HTs	Hydrolysed Tannins
CTs	Condensed Tannins
PA	Phytic Acid
NSPs	Non-Starch Polysaccharides
FG	Free Gossypol
OZT	Oxazolidinethione
ITC	Isothiocyanate
EB	Electron Beam
GR	Gamma Ray
ADF	Acid Detergent Fibres
